# COMBINED AND INTERACTIVE EFFECTS OF GLOBAL CLIMATE CHANGE AND TOXICANTS ON POPULATIONS AND COMMUNITIES

**DOI:** 10.1002/etc.2045

**Published:** 2013-01

**Authors:** S Jannicke Moe, Karel De Schamphelaere, William H Clements, Mary T Sorensen, Paul J Van den Brink, Matthias Liess

**Affiliations:** † Norwegian Institute for Water ResearchOslo, Norway; ‡ Laboratory of Environmental Toxicology and Aquatic Ecology, Ghent UniversityGhent, Belgium; § Department of Fish, Wildlife, and Conservation Biology, Colorado State UniversityFort Collins, Colorado, USA; ‖ EnvironAtlanta, Georgia, USA; # Department of Aquatic Ecology and Water Quality Management, Wageningen UniversityWageningen, The Netherlands; †† Alterra, Wageningen University and Research CentreWageningen, The Netherlands; ‡‡ UFZ-Helmholtz Centre for Environmental ResearchLeipzig, Germany

**Keywords:** Ecological risk assessment, Stressor interaction, Population ecotoxicology, Community ecotoxicology, Cost of adaptation

## Abstract

Increased temperature and other environmental effects of global climate change (GCC) have documented impacts on many species (e.g., polar bears, amphibians, coral reefs) as well as on ecosystem processes and species interactions (e.g., the timing of predator–prey interactions). A challenge for ecotoxicologists is to predict how joint effects of climatic stress and toxicants measured at the individual level (e.g., reduced survival and reproduction) will be manifested at the population level (e.g., population growth rate, extinction risk) and community level (e.g., species richness, food-web structure). The authors discuss how population- and community-level responses to toxicants under GCC are likely to be influenced by various ecological mechanisms. Stress due to GCC may reduce the potential for resistance to and recovery from toxicant exposure. Long-term toxicant exposure can result in acquired tolerance to this stressor at the population or community level, but an associated cost of tolerance may be the reduced potential for tolerance to subsequent climatic stress (or vice versa). Moreover, GCC can induce large-scale shifts in community composition, which may affect the vulnerability of communities to other stressors. Ecological modeling based on species traits (representing life-history traits, population vulnerability, sensitivity to toxicants, and sensitivity to climate change) can be a promising approach for predicting combined impacts of GCC and toxicants on populations and communities. Environ. Toxicol. Chem. 2013;32:49–61. © 2012 SETAC

## INTRODUCTION

Understanding and predicting the effects of chemical stressors on populations, communities, and ecosystems are a primary focus of ecotoxicological research. Demographic alterations in populations, structural changes in communities, and functional responses of ecosystems following exposure to a variety of chemical stressors are well documented in the literature 1. The consideration of basic ecological principles and mechanisms has greatly improved our ability to understand and predict such responses 2, although major challenges remain for a true integration of ecotoxicology into stress ecology 3. One such challenge is to deal with the complexity of multiple stressors in natural environments, where chemical stressors are only one of many components. In this context, the importance of global climate change (GCC) and its potential interactions with contaminants in the environment has received more attention recently 4–6. However, the implications of GCC in the ecological risk assessment (ERA) of chemicals still need to be evaluated. With this background, the Society of Environmental Toxicology and Chemistry (SETAC) convened a workshop (July 2011) to address a variety of issues associated with the potential impacts of GCC on chemical fate, exposure, toxicity, and risk assessment 7.

**Fig. 1:**
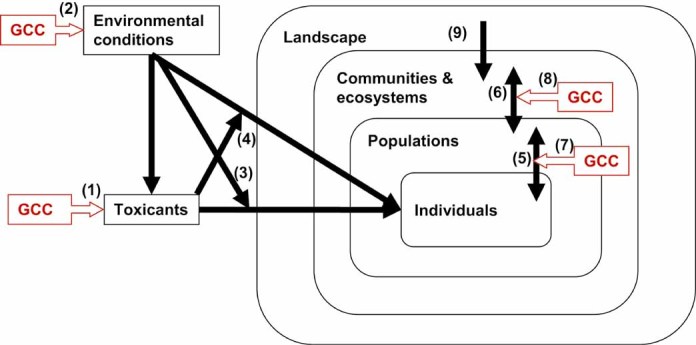
We address combined impacts of global climate change (GCC) and chemical stressors across biological levels of organization in the following way: The term GCC represents climatic drivers (temperature, precipitation, etc.). Environmental conditions represent other abiotic factors (hydrologic regimes, ultraviolet radiation, nutrient concentrations, etc.). Global climate change can affect the fate and exposure of toxicants directly (arrow 1) or through altered environmental conditions (arrow 2) 9. Individuals can be impacted by GCC-related changes in toxicant exposure and/or other environmental conditions; interactions between these factors can result in climate-induced toxicant sensitivity (arrow 3) or toxicant-induced climate-sensitivity (arrow 4) 13. When the combined toxicant and GCC impacts on individuals propagate higher levels, they can be modified by population-level (arrow 5) and community-level (arrow 6) processes. Such population- and community-level processes can in turn be impacted by GCC, directly or indirectly (arrows 7 and 8). Finally, landscape properties may influence the responses of populations and communities to combine toxicant and GCC effects (arrow 9). [Color figure can be seen in the online version of this article, available at http://wileyonlinelibrary.com]

Global climate change projections for the end of this century by the Intergovernmental Panel on Climate Change predict changes in a range of environmental conditions, such as higher mean temperature, change in precipitation patterns, higher ocean acidity, and reduced sea-ice cover 8 (see also an overview in Table 1 of Gouin et al. 9, in this issue). These projections include high uncertainties and regional variation; but, in general, increased frequency in extreme weather events such as heat waves, droughts, and storms is expected. The release, fate, and exposure of toxicants are also expected to be affected by GCC, through altered degradation rates 5, for instance, although there is still high uncertainty associated with GCC effects on future concentrations of contaminants in the environment 9. Moreover, societies' adaptation to GCC is also anticipated to affect pollution regionally by, for example, altered agricultural practices 10, increased pressure of pests and related pesticide application 11, and increased exploitation of polar regions 12. Even without any change in the levels of toxicant exposure, GCC-induced changes in other environmental conditions may also affect the sensitivity of organisms to current toxicants concentrations 13. Responses of individual organisms, however, are not necessarily predictive of impacts on higher levels of biological organization such as populations and communities 3, 14. These levels usually form the basis of our ecological protection and restoration goals 15. In the present study, therefore, we consider the combined effects of GCC and toxicants at the population and community levels (Fig. 1). The ultimate aim is to provide support for improved ERA 16 and ecosystems restoration 17 under GCC.

The combination of toxicant stress with other environmental stressors, such as heat stress or desiccation, can often result in a stronger-than-additive effect on organisms 18. Nonadditive stressor interactions are particularly important in population- and community-level ERA because they complicate extrapolation and forecasting of impacts. If effects of a toxicant will be more pronounced under future climatic conditions, more stringent environmental quality standards will be needed for this chemical. Regarding ecological restoration 17, removing one stressor may result in a greater benefit than expected in case of a synergistic interaction, while in case of an antagonistic interaction, removing one stressor may be less effective than expected. The physiological mechanisms underlying the interactive effects of toxicant and climatic stressors can be interpreted from two different angles, as proposed by Hooper et al. 13: (1) climate-induced toxicant sensitivity (CITS), where exposure to a climate-related stressor makes an organism more sensitive to subsequent toxicant exposure (Fig. 1, arrow 3), and (2) toxicant-induced climate susceptibility (TICS), where toxicant exposure makes an organism more vulnerable to subsequent changes in climatic conditions (Fig. 1, arrow 4). Here, the term synergistic interaction will also be used in the statistical sense (i.e., any stronger-than-additive effect of two stressors), even if the underlying mechanism is unknown.

In the present study, we are concerned with how combined impacts of climate and toxicant stress measured for individual-level responses propagate to the population level (e.g., population growth rate) and ultimately to the community level (e.g., species diversity and ecosystem functions and services). Unfortunately, most studies of interactions between GCC-related and toxicant stress factors have dealt only with individual-level responses (e.g., survival, development, reproductive rates). A review of multiple environmental stressors' effects across levels of biological organization 19 suggests that interaction types (synergistic, additive, or antagonistic) vary with the specific stressor combination, the trophic level (e.g., herbivores vs predators), and the response level (population vs community). Compounded stressors are thus likely to yield ecological surprises in real ecosystems 20. This means that it will be difficult to make general predictions about how individual-level responses to climate and toxicant stressors will propagate to higher levels. The adverse outcome pathway approach used for predicting multiple stressor effects at the individual level 12 may to some degree be applicable for extrapolation to the population level (e.g., population growth rate) but not necessarily to the community level, where species interactions must also be considered.

In the present study, instead, we focus on ecological mechanisms operating at the population and community levels, which may contribute to either compensation or exacerbation of individual-level effects of stressors at the higher levels of biological organization (Fig. 1, arrows 5 and 6). We consider ecological mechanisms affecting both short-term and long-term responses to stressors, as well as the role of spatial dynamics and landscape structures (Fig. 1, arrow 9). We must also consider the many potential impacts of climate change on population and community processes 21 (Fig. 1, arrows 7 and 8). Many recent reviews have argued that GCC is or will be affecting communities and ecosystems 22–24, for example, through changes in phenology (timing of events 25), species range boundaries 26, species invasions 27, species interactions 28, and increased extinction rates 29. Although some species may benefit from higher temperature and other GCC-related changes, a large number of species are expected to be vulnerable to impacts of climate change 10. In the present paper, therefore, we address two key questions: (1) How will GCC-related changes in environmental conditions affect the vulnerability of populations and communities to toxicants? (2) How will combined impacts of toxicants and GCC-related stressors at the individual level be influenced by ecological mechanisms at the population and community levels?

We focus on ecological mechanisms grouped into these four topics: (1) demographic and interspecific processes influencing propagation of individual-level responses; (2) resistance, resilience, and recovery from disturbances; (3) acquired tolerance to stressors and associated costs; and (4) species traits and population vulnerability in a landscape context.

**Fig. 2:**
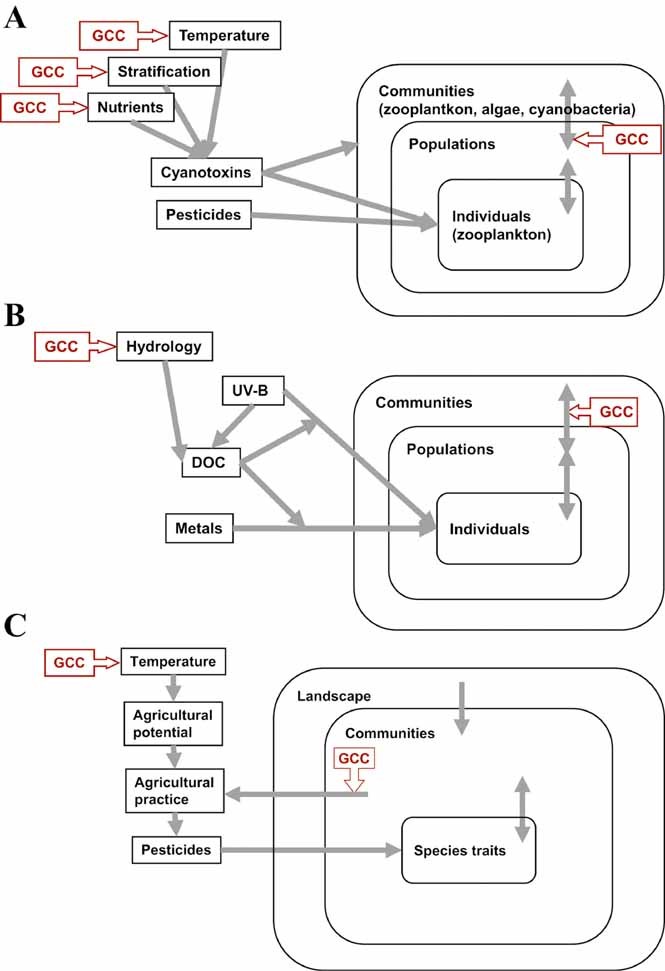
Illustration of case studies, based on Figure 1. For each case study, the figure indicates global climate change (GCC)–related changes in environmental conditions that may affect the vulnerability of populations and communities to toxicants and ecological processes affecting the population- and community-level responses. More details on each case study are given in the main text. (**A**) *Case study 1*: Impacts of global warming and cyanotoxins on plankton communities in lakes. (**B**) *Case study 2*: Impacts of ultraviolet (UV) radiation and metals on invertebrate communities in streams. (**C**) *Case study 3*: Impacts of future climate-related pesticide use on invertebrate communities in streams. [Color figure can be seen in the online version of this article, available at http://wileyonlinelibrary.com]

In the following section, we discuss ecological mechanisms operating at the population and community levels for each of these topics and give examples of how these mechanisms can influence responses to climate change and toxicants. For topics 2 through 4, we also present case studies describing relevant mechanisms in more detail (Fig. 2) and give suggestions for further research. Since few studies have yet examined combined responses to toxicant exposure and climatic change in natural populations and communities, the case studies are based on recent and ongoing research by the authors. The case studies focus on secondary environmental effects of climate change in different regions—that is, not only increased temperature but also changes in precipitation patterns, species interactions, and pesticide use. Finally, we recommend a set of research approaches with particular relevance for ERA and ecosystem restoration.

## DEMOGRAPHIC AND INTERSPECIFIC PROCESSES INFLUENCING PROPAGATION OF INDIVIDUAL-LEVEL RESPONSES

### Population level

It is well known that interactions among individuals need to be accounted for when extrapolating stressor effects from the individual level (i.e., intrinsic toxicant sensitivity) to the population level 30, 31. In natural populations, density-dependent processes such as competition for food can mitigate stressor impacts at the population level compared to the individual level. For instance, mortality due to toxicant exposure can reduce competition for food and thus reduce starvation. The resulting reduction in density-related mortality can to some degree compensate toxicant-related mortality 32, 33. In a similar manner, the response of a population to climatic stress factor may be modified by density-dependent mechanisms. In cases where density-dependent processes compensate for the impact of stressors, population-level impacts can be weaker than expected from the individual-level impacts. In contrast, other processes may result in a stronger response to climate and toxicant interactions at the population level than expected from the individual responses. These factors include sublethal and latent effects and the timing of multiple stressors in relation to the life-history stages. For example, it has been postulated that dryness associated with climate change may interact with contaminant exposure to accelerate amphibian declines 34. In a laboratory study 35, exposure of salamander (*Ambystoma barbouri*) larvae to ecologically relevant doses of the herbicide atrazine (40 and 400 µg/L) via the matrix did not result in mortality. Nevertheless, when the salamanders reached the postmetamorphic stage, the atrazine exposure eight months earlier caused weight loss and higher risk of desiccation. The mechanisms for these effects are unknown, but Rohr and Palmer 35 suggested that atrazine may have altered neuroendocrine development associated with the expression of water-conserving behaviors. Regardless of the mechanism, atrazine clearly affected behaviors tied to water retention. Moreover, the postmetamorphic life-history stage is particularly important to the population dynamics of amphibians. Thus, in natural populations, the combination of atrazine exposure in the larval stage and drought in the adult stage may result in a synergistic interaction, which is difficult to predict from individual-level responses in laboratory studies 35.

Another example of complex interactions between chemicals and climatic conditions is the combined effects of daily maximum temperature and mercury (Hg) exposure on nestling production in tree swallows (*Tachycineta bicolor*) 36. During the early nestling stage, the combined effect of Hg exposure and temperature was an antagonistic interaction: at the reference site, higher maximum temperature (increase by ∼5–6°C) increased nestling production (on average from about four to five individuals), while at Hg-contaminated sites, increased maximum temperature resulted in lower nestling production (from about four individuals to two to three individuals). In the late nestling stage, however, there was no interaction between the two variables: temperatures were positively related to nestling production in both types of sites (see de Hoop et al. 12 for more details on the physiological mechanisms). Understanding how temperature affects responses to toxicants in different life-history stages will be relevant for ERA at the population level.

The examples given above illustrate the difficulty of making general statements on how climate and toxicant interactions may propagate from the individual to the population level. However, the following issues can be important to address for risk assessment for a given population: (1) the combined impact of climate and toxicant stress—experienced sequentially as well as concurrently—in the different life-history stages; (2) the population's capacity for compensating the combined stress by density-dependent mechanisms in the different stages; and (3) the sensitivity of a population's growth rate to changes in demographic rates in the different stages.

### Community level

Because climate change is predicted to have profound impacts on species interactions and food-web structures 23, 28, we can expect that the combined effect of climate change and toxicants will yield complex results at the community level. So far, there are few published examples of climate and toxicant interactions that also involve species interactions. A recent study on Western Hudson Bay polar bears (*Ursus maritimus*) demonstrates that recent changes in the timing of sea-ice breakup correlate with a diet change from ice-associated seal species to more open water–associated seal species 37. The open water–associated seal species tend to have higher tissue concentrations of chlorinated and brominated contaminants (for more details on physiological mechanisms, see Hooper et al. 13). This climate-induced shift in predator–prey interactions has therefore resulted in increased contaminant concentrations in the polar bears 37. In contrast, tawny owls (*Strix aluco*) accumulated more PCB in years with colder winters than in years with warmer winters. One of the proposed explanations was that in strict winters, availability of the preferred prey (voles) was restricted, and the owls may have been forced to feed on alternative prey (passerine birds) with higher contaminant loads 38. Clearly, more knowledge is needed on the impacts of GCC on trophic interactions for better prediction of toxicant exposure and bioavailability through food webs 39, 40.

The review of Crain et al. 19 suggests that interactive effects of multiple stressors at the community level are more commonly antagonistic than synergistic, which implies that interspecific interactions may more often compensate than enhance multiple stressor effects. Others have concluded the contrary, that multiple stressors at the community level are more commonly synergistic 20. At any rate, none of the multiple stressor studies reviewed in these articles involve the combination of GCC and toxicants. To our knowledge, no experimental studies have analyzed the combined impacts of climate and toxicant effects on species interactions and community-level end points. A combination of factorial studies with the use of mechanistic community models 41 is recommended to address the above research questions. More generally, important research questions to be addressed include the following. First, it is important to investigate what kind of species interactions will be most susceptible to climate change. Examples already mentioned include decline of top predators and specialist feeders, temporal decoupling of synchronized species interactions (e.g., predator–prey or pollinator–plant), shift of species range and species invasion, and increased occurrence of pest species, parasites, and diseases. Second, research should focus on how may these changes in species interactions affect sensitivity to toxicants for various species in the community and eventually the overall community-level responses.

## RESISTANCE, RESILIENCE, AND RECOVERY FROM DISTURBANCES

### Population level

Recovery of affected populations following episodic exposure to a stressor is governed by many factors such as the persistence of the stressor, type of stressor, time of year when it occurs, distance to nonstressed habitat with recolonization sources, and species traits related to life history and dispersal 42, 43. Effects of GCC on the persistence of compounds will be both chemical- and region-specific. The recovery potential of affected populations may also be influenced by climate change 44, both directly (by temperature increase) and indirectly (by change in other environmental conditions, e.g., increased eutrophication). Recovery times of a zooplankton (Cladocera) population after chlorpyrifos exposure in a microcosm experiment in a temperate climate were not directly affected by temperature increase, but they increased with nutrient additions 45. A similar mesocosm experiment performed in the Mediterranean region showed that the time required for full recovery of the affected zooplankton populations was longer than in corresponding experiments performed in temperate regions 46. The role of higher temperature as well as indirect effects, such as algal blooms, seemed to be critical factors that determined recovery times. This increase in recovery times from temperate to Mediterranean conditions was attributed to the increase in temperature being nonoptimal for zooplankton and beneficial for digestion-resistant and toxic phytoplankton species, for example, cyanobacteria 47. Hence, it may be expected that GCC contributes to prolonged recovery in areas where episodic increases in temperature co-occur with exposure to chemical stressors and where nutrient concentrations are high enough to stimulate cyanobacterial blooms (see also *Case study 1*). A combination of controlled factorial experimental studies with mechanistic ecosystem models can help to more precisely delineate those combinations of temperature and nutrient levels that may affect the recovery time of populations from chemical exposure.

On larger spatial scales, landscape features and the functional connectivity of recolonization sources are of key importance for the recovery of local populations from toxicant exposure. Spromberg and Scholz 48 modeled the impact of prespawn mortality and dispersal rates of coho salmon (*Oncorhynchus kisutch*) populations in the greater metropolitan area of Seattle, Washington, USA, using a metapopulation model (i.e., a set of populations connected by dispersal of individuals) and varying dispersal rates. Prespawn mortality (PSM) is largely caused by toxic urban storm water runoff. At lower dispersal rates, PSM–affected populations were more likely to experience local extinction due to higher isolation. Higher dispersal rates provided more emigrants from nonaffected source populations and maintained PSM–affected populations at higher abundances. However, the larger dispersal connecting the PSM–affected populations and the nonaffected source population came at a cost in terms of reducing the total metapopulation abundance. In this system, the dispersal behavior of the salmon populations will clearly have high importance for the toxicant impacts and recovery both locally and regionally. The runoff of toxic storm water is correlated with fall patterns of rainfall, which may be impacted by the climate change projected for this region. However, because PSM depends on many environmental factors and their timing, it is not yet possible to predict how climate change will affect toxicant exposure, mortality, and recovery in this metapopulation. More generally, climate change may in principle affect the presence and functional connectivity of recolonization sources, but such effects will depend on the region and the species in question. Research in this regard should focus on combining metapopulation modeling with predictions of expected spatial distributions of recolonization sources under different GCC scenarios.

### Community level

**Fig. 3:**
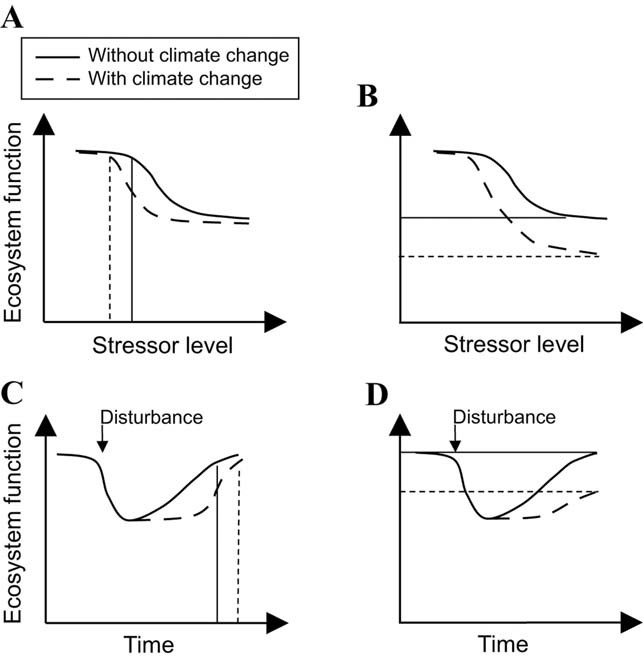
The hypothesized influence of global climate change (GCC) on community responses to toxicants. Ecosystem function represents the community-level end point. (**A**) Communities subjected to GCC are expected to have lower resistance to other stressors; a threshold response will therefore occur at a lower stressor level. (**B**) Furthermore, various ecological mechanisms can result in GCC and toxicant interactions at the community level, here illustrated as a synergistic interaction. (**C**) Lower resilience in communities subjected to GCC implies that recovery from a disturbance will take more time (or higher restoration effort). (**D**) Climatic and/or toxicant stress can have lasting effects on community composition and prevent it from returning to its former state after a disturbance; ecological positive feedback mechanisms may further keep the community trapped in an alternative state.

The theoretical concepts of community resistance and resilience (see Newman and Clements 1) are useful for considering a community's response to and recovery from a chemical stressor under climate change. *Community resistance* represents the magnitude of disturbance that a community can tolerate before being pushed to a different state. In contrast, *resilience* represents the rate or time of return to predisturbance conditions. Disturbed communities will often experience alteration of species composition, loss of species, and reduced functional redundancy, which increases the susceptibility of these communities to other perturbations (Fig. 3A and B) (many examples are given by Paine et al. 20). As described above, most of the available evidence indicates that populations and communities will be impaired by climatic changes such as increased temperature or greater hydrologic variability. Although some species may benefit from such changes, the overall effect on communities will likely be the elimination of sensitive species, lower diversity, and loss of functional redundancy. Moreover, the remaining species in affected communities may be pushed to the limits of their distribution range or experience less optimal conditions. These communities are likely to show lower resistance to additional disturbances such as contaminant exposure and slower recovery after contaminant exposure (i.e., lower resilience; Fig. 3C and D). Impaired resistance and/or resilience is particularly serious for communities that exhibit a threshold-type response to a stressor 20, 49, that is, abrupt changes in community structure or function as a consequence of small increases in stressor level. Thresholds in responses to environmental stressors have been identified in numerous ecosystems, such as Arctic food webs 24, polluted coastal ecosystems, and eutrophic lakes 50. In ecosystems with threshold-type responses to stressors and positive feedback mechanisms, a perturbation exceeding the threshold level may shift the community structure to an alternative stable state 51 (Fig. 3D). Due to the feedback mechanisms, an alternative state may remain stable long after the stressor is removed, and restoring a system to predisturbance conditions may require that stressor levels are reduced significantly below those that triggered the initial response 51.

Reduced resistance as a result of climate change may narrow the presumed safe level of a stressor and cause threshold shifts to occur at a lower level (Fig. 3). For example, coral reefs in the Caribbean and the Great Barrier Reef subjected to the combined effects of impaired water quality (runoff of nutrients and sediment from land) and increased temperature have abruptly shifted from healthy, diverse communities to barren rock outcrops dominated by sea urchins 49. The resilience of shallow lake communities can also be impaired by GCC: the risk that lakes subjected to excessive nutrient loads will shift from a clear, macrophyte-dominated state to a turbid, phytoplankton-dominated state with higher risk of harmful algal blooms is expected to increase with global warming 52 (see *Case study 1*). We postulate that communities with reduced resistance and/or resilience due to climate change will have a higher risk of exceeding a threshold in response to toxicant stressors, in a similar way as reported for other abiotic stressors 49, 52, and potentially reach an alternative state.

### Case study 1: Impacts of global warming and cyanotoxins on plankton communities in lakes

#### Scenario of climate change and toxicant exposure

This case study represents lakes subjected to both pesticides and eutrophication (nutrient enrichment) in general. Cyanobacterial harmful algal blooms (cyanoHABs) are proliferating in lakes and coastal waters worldwide due to increased nutrient inputs and are a considerable threat to ecosystem quality 53 as well as to ecosystem services such as use of water resources for consumption and recreational purposes. Many cyanobacteria species are able to produce toxins, generate hypoxia, and alter food webs. In this case study, therefore, cyanobacteria represent both an indirect GCC impact and a community component that interacts with other species (Fig. 2A). Climatic changes play an important role by increasing the frequency, intensity, geographic distribution, and duration of cyanoHABs 53. Climatic warming is likely to favor cyanoHABs because they exhibit optimal growth rates at relatively high temperatures, usually above 25°C, at which they compete most effectively with other phytoplankton (i.e., eukaryotic algae) 52.

#### Combined impacts of climate change and toxicants on communities

We predict that the effects of higher temperature on the formation of cyanoHABs may be exacerbated by the presence of toxicants such as pesticides, resulting in an interactive effect of (indirect) GCC impacts and toxicants on aquatic communities. Our prediction is based on the following experimental evidence, combined with theoretical considerations of species interactions within communities. Firstly, a warmer climate will favor the growth of cyanobacteria over eukaryotic algae, resulting in a higher risk of the occurrence of cyanoHABs with toxic impact on zooplankton 53. Secondly, exposure of zooplankton to pesticides can result in lower grazing pressure on phytoplankton (due to increased zooplankton mortality, for example), leading to increased phytoplankton biomass (including cyanobacteria), as found in mesocosm studies 3. Thirdly, the cyanotoxins may reduce the resistance of the zooplankton community, resulting in a greater vulnerability to pesticides. This combination of stressors may even result in synergistic effects for individual zooplankton, as reported for *Daphnia pulicaria* exposed to both cyanobacteria and carbaryl 54. Fourthly, cyanotoxins may contribute to increased recovery time of zooplankton from pesticide exposure, as demonstrated for *Daphnia* spp. exposed to carbaryl in mesocosms 47. In other words, increased temperature may indirectly—due to increased risk of cyanoHABs—contribute to reduced resistance and resilience of the zooplankton community to other stressors.

#### Relevance for ERA and suggestions for further research

It has already been stated that water managers will have to accommodate the effects of climatic change in their strategies to combat the expansion of cyanobacterial blooms 52. In this respect, managers may wish to address the question of synergistic stressor impacts: Will the increase of cyanoHABs and their impacts on communities under both temperature increase and pesticides combined be higher than the expected increases caused by temperature increase and pesticides separately? Mesocosm studies have revealed interactions of different toxic stressors at the community level 55 and may therefore be an ideal tool for addressing the above question. Ecosystem modeling may in this regard be useful to generate testable hypotheses for different pesticides and their interaction with temperature effects, together with more experimental data on the combined effects of cyanotoxins and chemical contaminants on individual zooplankton.

## ACQUIRED TOLERANCE TO STRESSORS AND ASSOCIATED COSTS

### Population level

**Fig. 4:**
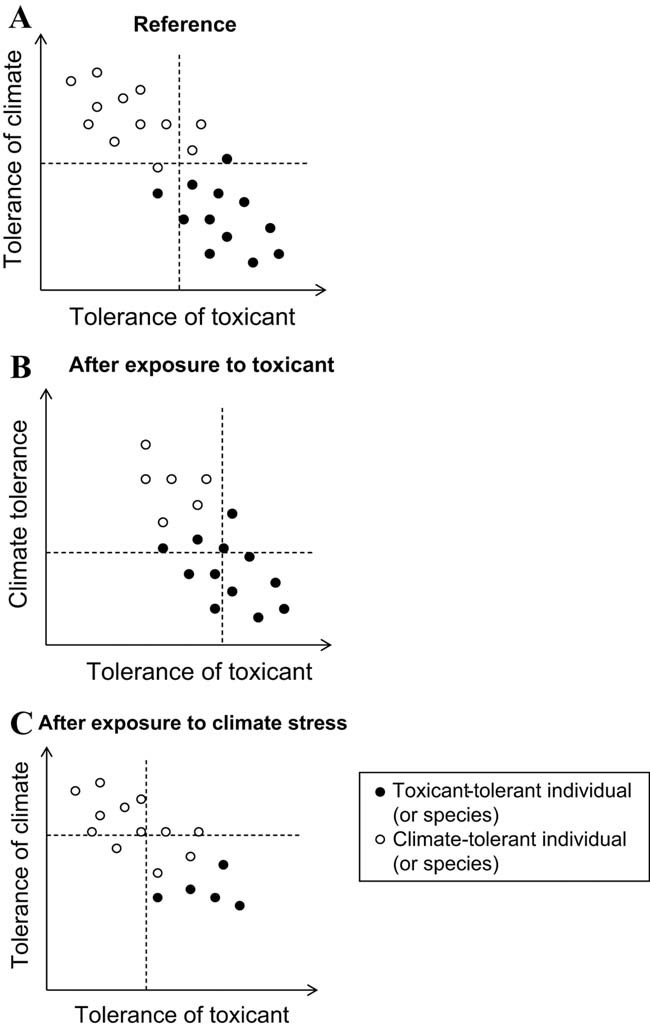
Simplified visualization of the cost of tolerance concept. (**A**) The points represent individuals within a population. Individual differences in genetic makeup result in variable tolerance of climate (i.e., a stressor related to GCC) and tolerance of a toxicant. A genetic trade-off between the two types of tolerance exists within the population. Individuals can therefore be categorized as either climate-tolerant or toxicant-tolerant. The dashed lines represent the population average for these two traits. (**B**) Following toxicant exposure over multiple generations, natural selection will favor the more toxicant-tolerant individuals; therefore, the population's average tolerance of the toxicant will increase. The average tolerance of climate will consequently decrease. In addition, the overall genetic diversity will decrease for both traits. (For simplicity, the recruitment of new individuals is not included in the illustration.) (**C**) Conversely, during long-term climatic stress, natural selection may favor the more climate-tolerant individuals and thereby reduce the average toxicant tolerance of the population (as well as genetic diversity). The cost of tolerance concept can also be applied to the community level; the points then represent different species within a community.

When a population is exposed to a stressor over multiple generations, natural selection may favor genotypes that are more tolerant to this stressor and thereby gradually increase the mean tolerance of the population (Fig. 4). Many studies have demonstrated local adaptation (acquired genetic resistance) of field populations with a history of stress exposure, for both chemical contaminants 56 and climate-related factors 22.

A potential disadvantage of genetic adaptation to a chemical stressor is that acquired tolerance can be associated with reduced fitness in the absence of that stressor (although there are also examples of increased fitness or no fitness cost). For example, an oligochaete population (*Limnodrilus hoffmeisteri*) evolved tolerance to cadmium (Cd) in a metal-contaminated site in the Hudson River (New York, USA); however, following a remediation of this site, the tolerance was lost after 9 to 18 generations 57. This rapid loss of tolerance was probably due to a trade-off between adaptation to Cd and some life-history trait, such as somatic growth rate.

Genetic adaptation to a toxicant usually results in a reduction of genetic diversity at the population level 58. Populations in a contaminated environment can experience additional population-genetic processes due to reduced population size, such as bottlenecks and inbreeding depression, which can further contribute to genetic diversity loss and increased extinction risk. Furthermore, adaptation to one set of environmental stressors may also increase the susceptibility to different environmental stressors (Fig. 4). Freshwater snails (*Biomphalaria glabrata*) exposed to Cd (0.05–1 µM) continuously for three generations showed latent costs of adaptation, expressed as decreased tolerance to temperature stress (increase from 26 to 36°C) in the fifth generation 59. A population of Atlantic tomcod (*Microgadus tomcod*) in the Hudson River developed resistance to PCB during 30 years of exposure to this chemical 60. The authors believe that the resistance to PCB acquired by tomcod is likely to be accompanied by evolutionary costs in terms of heightened sensitivity to other stressors, such as PAHs.

Considering adaptation to chemical stressors under climate change, the following two questions are highly relevant for ERA: (1) If a population has genetically adapted to long-term chemical contamination, will it exhibit a lower tolerance and/or a lower ability to adapt to future climatic changes? (2) If a population is able to genetically adapt to current or future climate change, will it show a lower tolerance and/or a lower ability to adapt to future chemical stressors? The first question is important for assessment of future ecological impacts of climate change on currently or historically contaminated locations, even after the contamination exposure has terminated. The second question is important for prospective risk assessment of new chemicals under future climatic scenarios. These two types of population-level trade-offs are analogous to the individual-level trade-offs described by Hooper et al. 13 as TICS and CITS, respectively (Fig. 1); the population-level trade-off involves population-genetic mechanisms in addition to physiological mechanisms. Available data on genetic correlations between tolerance to toxicants and to climate change (i.e., trade-offs) at the population level are very scarce 61. An important research question in this respect is which combinations of chemicals and climatic stressors are likely to result in unaltered tolerance (additivity), cost-of-tolerance (i.e., a synergistic interaction), or co-tolerance (antagonistic interaction) between chemical and climatic stressors.

### Community level

Research on the cost of acquired tolerance has mostly focused on populations 56, 62, but this concept is also relevant for community-level impacts of long-term stressor exposure. Similar to the way a population's genetic composition reflects the unique history of that population over evolutionary time, the species composition of a community reflects its unique history of disturbance. Pollution-induced community tolerance is relatively common in communities that have been exposed to a variety of stressors 63–65. In these cases, exposure to contaminants results in elimination of the most sensitive species and thereby a net increase in community tolerance to these stressors. However, pollution-induced community tolerance may involve a trade-off: the loss of sensitive species implies that the community has lower diversity and, therefore, lower probability of containing species with tolerance for novel stressors, including GCC-related stressors (Fig. 4). Experiments conducted with macroinvertebrate communities from a contaminated stream and a reference stream showed that metal-tolerant communities were more susceptible to novel stressors than reference communities 65–67 (see *Case study 2*). These results suggest that communities from chronically polluted environments may be at greater risk from subsequent influences of GCC (i.e., a community-level mechanism for TICS). Vice versa, communities that have obtained higher tolerance to GCC-related stress, for example, by elimination of drought-sensitive species, may in turn become more sensitive to novel chemical stressors (i.e., CITS).

### Case study 2: Impacts of ultraviolet radiation and metals on invertebrate communities in streams

#### Scenario of climate change and toxicant exposure

High-elevation streams in the southern Rocky Mountain ecoregion (Colorado, USA) provide a unique opportunity to investigate the combined effects of contaminants and GCC. Regional models of climate warming for the western United States predict significant decreases in precipitation, snowpack, and stream discharge and alterations in biogeochemical processes 68. For example, decreases in snowpack and stream discharge will reduce stream depth and decrease the input of dissolved organic carbon (DOC) exported to watersheds (Fig. 2B). Reduced stream depth and lower concentrations of DOC, which are the primary factors responsible for attenuating ultraviolet (UV) radiation in aquatic ecosystems, will result in greater UVB exposure, which may be harmful to benthic communities 69. In addition to the direct effects of GCC and UV, many of Colorado's high-elevation streams are subjected to heavy metal pollution from over 20,000 abandoned mines in the region. Because DOC reduces the bioavailability and toxicity of metals 70, reduced export of DOC to streams will increase the direct impacts of these contaminants.

#### Combined impacts of climate change and toxicants on communities

This case study is based on a long-term (1989–2010) research program focused on the upper Arkansas River, a metal-polluted stream in the southern Rocky Mountain ecoregion 71. Mining operations in this watershed since the mid-1800s have resulted in elevated concentrations of metals (Cd, Cu, and Zn). This monitoring program measured physicochemical characteristics, heavy metal concentrations, and macroinvertebrate community structure seasonally at locations upstream and downstream from several sources of metal contamination. Stream mesocosm experiments were also conducted to examine the combined effects of UVB and metals on benthic communities collected from reference and metal-contaminated sites. Results of this research demonstrated that penetration of UVB radiation to the benthos and concentrations of heavy metals and DOC were closely associated with temporal variation in stream discharge 69, 72. Relatively modest (25%) reductions in stream depth and DOC as a result of climate-induced changes in stream hydrology could double UVB penetration to the streambed. Results of stream mesocosm experiments conducted with communities from the Arkansas River provided support for the hypothesis that organisms from chronically polluted streams were more susceptible to UVB radiation, compared with those from unpolluted streams 66. Consistent with predictions of the pollution-induced community tolerance hypothesis, communities from metal-polluted sites were more tolerant of heavy metals, compared with naive communities. The underlying mechanism can be operating on the population level (selection of tolerant individuals) as well as on the community level (selection of tolerant species). Communities from metal-polluted sites were also more sensitive to UVB radiation, which indicates TICS. These experiments also demonstrated that effects of UVB radiation on stream metabolism, a functional measure associated with ecosystem production, were greater for metal-polluted communities, compared with reference communities. Overall, these findings demonstrate that exposure of stream benthic communities to UVB radiation will likely increase as a result of GCC and that superimposing UVB on chronically contaminant-disturbed communities may result in a cost-of-tolerance response at the community level.

#### Relevance for ERA and suggestions for further research

The acquired tolerance reported in this case study is also likely to impact the resilience of the communities. However, recovery of the macroinvertebrate communities following restoration is difficult to quantify accurately even for this well-studied ecosystem, because recovery of these communities is influenced by many factors including seasonal episodic events 71. This case study demonstrates the importance of long-term monitoring of community composition for studying the impacts of multiple environmental stressors and assessing recovery. Existing long-term data from chronically polluted sites can be valuable for testing acquired community-level tolerance in other ecosystems and for assessing the impact on the communities' vulnerability to climatic changes and other perturbations.

## SPECIES TRAITS AND POPULATION VULNERABILITY IN A LANDSCAPE CONTEXT

### Population level

In ecotoxicology, there is growing interest in linking population vulnerability to chemical stress by means of the species-trait approach 73, which can also be useful for considering impacts of climate change. Rubach et al. 74 recently presented a framework showing how life-history traits of species can be quantitatively related to the parameters of classical ecotoxicological models. In this framework, population vulnerability (i.e., the likelihood of a species becoming locally extinct) is defined as the product of three components—external exposure, intrinsic sensitivity, and population sustainability. For each of these vulnerability components, relevant traits have been identified as follows: external exposure depends on a species' food choice and active avoidance; intrinsic sensitivity depends on many traits including assimilation efficiency, toxicant elimination ability, toxicant sequestration, and biotransformation potential; and population sustainability depends on several traits related to recolonization, recovery, and dispersal ability.

The intrinsic sensitivity of species to toxicants can be considerably influenced by change in climatic variables such as temperature 5, 18, 75. The majority of laboratory toxicity tests (with individual-level end points) report that increased temperature leads to higher intrinsic sensitivity, although there are also examples of the opposite response or even no relationship 75, 76, 13. For natural populations, however, the role of temperature in intrinsic sensitivity to toxicants must be considered in the context of the climatic region to which they are adapted. Comparison of the sensitivity of species from different geographical and climatic regions, based on species sensitivity distributions, showed no consistent differences between species from different parts of the world 77. Likewise, comparison of species sensitivity distributions for polar and temperate marine organisms to oil components showed no difference in sensitivity across these climatic regions 12. These results suggest that current regional differences in climatic conditions do not systematically influence the intrinsic toxicant sensitivity of species in their natural environment. Instead, the toxicant sensitivity of a species is more likely to be impacted by changes from current climatic conditions, such as increased temperature and increased occurrence of heat waves.

Potential impacts of GCC on sensitivity to toxicants can only be tested experimentally for a very limited number of species. An alternative way forward for assessing the vulnerability of species to this combination of stressors may be based on the identification of traits associated with more general vulnerability to climate change. A recent assessment by the European Environment Agency 10 of vulnerability to climate change for a large number of rare or potentially threatened species in Europe (birds, reptiles, amphibians, butterflies, and vascular plants) showed that greater vulnerability is often associated with limited dispersal abilities. More specific traits that can be associated with vulnerability to climate change have been identified within freshwater invertebrate species groups. Hering et al. 78 identified five key traits to describe sensitivity to climate change (increased temperature and associated changes in ecological niches) of Trichoptera (caddisfly) species: endemism, preference for springs, preference for cold water, short emergence period, and restricted ecological niches in terms of feeding types. Poff et al. 79 identified seven traits that relate benthic community composition successfully to, among other factors, climatic and hydrologic variables: voltinism (number of generations per year), stenothermy (sensitivity to temperature change), occurrence in drift, habit, rheophily (preference for running water), female dispersal ability, and resistance to desiccation. Based on these studies, we notice that traits associated with high intrinsic population growth rate and dispersal ability, the so-called r strategy, seem more robust to climate change. Conversely, traits more associated with the so-called K strategy (ability to compete successfully for limited resources in stable environments) might make these species more vulnerable to climate change. Hence, traits associated with low population sustainability in the framework of Rubach et al. 74 (low recolonization, recovery, and dispersal ability) are also likely to be associated with high vulnerability to GCC. For building on this framework to incorporate vulnerability to climate change, a key research question is whether traits associated with climate change vulnerability will overlap with traits associated with intrinsic toxicant sensitivity.

### Community level

The use of species traits is also an efficient concept to handle the complexity of assessing multiple stressor effects at the community level. Associations between species traits and various environmental factors have been identified over large geographical regions 80. A practical realization built on the associations between species traits and environmental factors is the species at risk (SPEAR) concept 2. Traits such as toxicological sensitivity, duration of life cycle, and migration have proven to link toxicant exposure to changes in community composition observed in natural streams. Using this approach, one may identify impacts of agricultural pesticides based on the trait composition of stream communities. Conversely, impacts on stream communities can be predicted based on knowledge about pesticide input. The latter has great potential for predicting the impacts of future GCC-linked increases in agricultural pesticide use (see *Case study 3*).

**Fig. 5:**
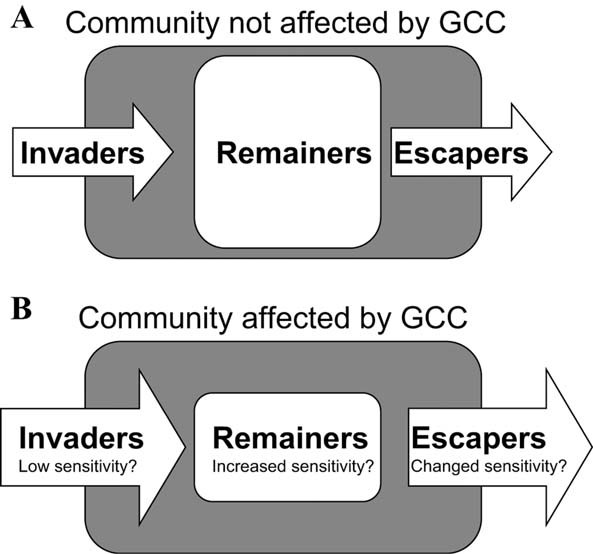
Conceptual model of impacts of global climate change (GCC) on species composition and toxicant sensitivity for a hypothetical community in a given location. (**A**) Prior to GCC impacts, the numbers of invaders and escapers are low relative to the number of remainers. (**B**) Due to altered environmental conditions, GCC is expected to increase the number of both invaders and escapers. We hypothesize that remainers that are unable to relocate toward favorable environmental conditions will experience more stress and, thus, increased sensitivity to toxicants (see *Species Traits and Population Vulnerability in a Landscape Context* for details).

Because it is expected that GCC will cause substantial shifts in spatial and temporal species distributions 10, 81 and thereby the trait composition of communities, we expect that GCC will also alter the community vulnerability to toxicant exposure. In this context, we suggest the new conceptual model invader/remainer/escaper (IRE; Fig. 5), which characterizes three groups of species within a community with different population-dynamic responses to climate change. The IRE concept suggests that in a location not yet affected by climate change (Fig. 5A), species are relatively well adapted to the local environmental conditions and most of the individuals of a given species tend to remain within the community. Consequently, there is little room for potential invaders in that area. Likewise, individuals in adjacent locations have a tendency to remain in their area, and the pressure of invasive species is relatively low. Under climate change (Fig. 5B), we expect that the local environmental conditions will increasingly deviate from the conditions to which the species are adapted, with implications for spatial population dynamics, community composition, and, indirectly, toxicant sensitivity.

In terms of population dynamics, it can be expected that some populations in a given location now show an increased rate of emigration, to escape from impaired conditions and migrate to new areas with more favorable climatic conditions (e.g., higher altitude). At the same time, escapers from other communities affected by climate change in adjacent areas (e.g., lower altitude) may migrate into the given location. Escapers that are able to settle successfully in this area will eventually become invaders in this community. It is expected that GCC will increase the number and severity of species invasions 27, and the IRE model illustrates three contributing factors (Fig. 5): (1) increased immigration rate due to suboptimal conditions in adjacent areas; (2) suboptimal environmental conditions for remainers in the area, which may reduce competitive ability relative to invaders; and (3) increased emigration rate from the given area, which leaves more resources and less competition pressure for invaders.

The demographic processes described in the IRE concept and the resulting changes in community composition are closely related to species traits. For example, species that are typical K strategists (high longevity, long generation time) have low dispersal capacity, are specialist feeders, and are more likely to be remainers. Remainers with a narrow temperature tolerance range (i.e., stenotherms) are particularly likely to experience stress due to GCC. Conversely, species with traits such as r strategists (high reproductive rate and short development time), which have greater dispersal ability and are feeding generalists, are more likely to colonize new areas in response to GCC and will thus more likely be escapers and potential invaders. Whether escapers from one area actually become successful invaders in a new area will depend on many factors in addition to the life-history traits. Physical barriers in the landscape may force potential escapers to be remainers and suffer from climatic stress. Moreover, species interactions and other stressors in the receiving community will influence the success of escapers in a new community. For example, the invasive snail species *Melanoides tuberculata* was able to displace the native *Biomphalaria glabrata* in spite of having a lower growth rate and fecundity, possibly because of its higher tolerance to both toxicants and climatic stressors (Cd, malathion, temperature extremes, and desiccation) 82. Populations that eventually become successful invaders may have a considerable impact on their new community.

In terms of sensitivity to toxicants, the IRE concept assumes that prior to climate change impacts, the toxicant sensitivity of remaining populations corresponded to the intrinsic toxicant sensitivity for the respective species 74. Following climate change, we hypothesize that remainers more often experience suboptimal environmental conditions and therefore have increased toxicant sensitivity (context sensitivity 83). We expect escapers to suffer less from GCC than remainers and thus to experience less increase in toxicant sensitivity than remainers, although the impact will depend on the environmental and ecological context of their new area. Populations with a high rate of escapers will also likely have reduced local abundance in their original area, which can impair the ability of these populations to recovery from toxicant stress. We expect the least increase in toxicant sensitivity for invaders, which by definition have settled successfully in a new area. The hypothesis of higher toxicant sensitivity for remainers versus escapers versus invaders can be tested in various ecosystems where climate-induced migration has occurred. If new research supports the IRE conceptual model, we propose that it could be used to identify species and populations for which GCC may most likely influence toxicant sensitivity and to assess the overall impact of community sensitivity to toxicants.

Finally, it is worth noting that GCC-induced species invasions can influence the toxicant sensitivity of their receiving community in multiple ways; altered species interactions may also influence the bioaccumulation of toxicants. In the San Francisco Bay delta, two years of climatic extremes (droughts followed by a flood) eliminated most of the native freshwater-intolerant species of the benthic community. These events probably contributed to the successful invasion of the nonnative Asian clam (*Potamocorbula amurensis*) 84. This species has permanently altered the community structure and trophic interactions and possibly increased the vulnerability of native fish and zooplankton populations by predation on eggs and larvae and by competition for food. Moreover, the invasion has caused increased selenium contamination in benthivore predators (sturgeon and diving ducks 85): selenium is strongly bioaccumulated in the Asian clam and may impair reproduction of species at higher trophic levels. This example illustrates the importance of considering spatial community dynamics (cf. the IRE concept) as well as ecological interactions for assessing interactions between climate change and toxicant impacts on communities. For a case study of ERA for the San Francisco estuary under climate change, see Landis et al. 16 in this issue.

### Case study 3: Impacts of future climate-related pesticide use on invertebrate communities in streams

#### Scenario of climate change and toxicant exposure

It has been postulated that changes in human activities in response to climate change (i.e., adaptation and mitigation) will have a larger impact on the quality of aquatic ecosystems than the direct climate change impacts will 86. Nevertheless, such indirect effects of GCC on freshwater ecosystems have rarely been studied. One example of a potential indirect GCC effect due to human adaptation is increase in pesticide runoff and its effect on aquatic communities. Shifts in the cultivation of certain crops toward higher latitudes, the reclamation of new areas, and the extension of cultivation periods can be expected 87. In addition, it is anticipated that global warming will lead to increases in the incidence of many insect pests through increased overwintering survival and longer seasonal activity and invasion of new pests. This will increase the pressure on agricultural production, which may in turn increase the application of pesticides 88.

#### Combined impacts of climate change and toxicants on communities

Kattwinkel et al. 11 assessed to what extent there may be indirect effects of GCC on freshwater communities due to changes in agricultural activities, using a species-trait modeling approach that also considered the role of landscape features (Fig. 2C). Potential exposure to insecticide (runoff potential) was predicted for current conditions (1990) and under a model scenario of future (2090) climate and land use, using a spatially explicit model on a European scale. Space-for-time substitution was used to predict future levels of insecticide application, intensity of agricultural land use, and cultivated crops. The key environmental characteristics of the near-stream environment influencing insecticide runoff in the model were topography, precipitation, soil type, soil organic carbon content, and the crops being cultivated. Runoff potential and landscape characteristics with relevance for the recovery of affected populations were combined to estimate the ecological risk of insecticides for freshwater communities, based on the indicator system SPEAR. The analysis also took into account the potential for recovery on the landscape scale: the presence of undisturbed upstream stretches that can act as sources for recolonization. Other potential climate change effects (e.g., pesticide decomposition and toxicity) were not included in this model. The model predicted a strong increase in the application of—and aquatic exposure to—insecticides under the future scenario, especially in central and northern Europe. This, in turn, would result in an increase in ecological risk for freshwater communities in these regions. The predicted increase in risk was particularly high in lowland areas of the Scandinavian and Baltic countries because these regions have a high potential for increased agriculture with higher temperature: the predicted proportion of affected streams in areas that included arable land increased from 6% (in 1990) to 19% (in 2090).

#### Relevance for ERA and suggestions for further research

The model predictions clearly illustrate the practical usefulness of a trait-based approach to identify areas that may become increasingly vulnerable to chemical stress under future climate change. The model predicted a strong impact of GCC on community-level effects of pesticides, even without accounting for more direct GCC effects on population vulnerability and community composition. Hence, the present study gives a first estimate of the likely direction of GCC-related changes in pesticide exposure and effect, which can be integrated with other GCC effects in future modeling work.

## CONCLUDING REMARKS AND RESEARCH RECOMMENDATIONS

We conclude that the four ecological topics discussed in this article are important both for interpreting existing observational data sets (see *Case study 2*) and for generating testable hypotheses regarding future effects of GCC and toxicant exposure (see *Case studies 1 and 3*). Nonetheless, these case studies are rather isolated examples, and there are few other published studies that address all these aspects of GCC and toxicants. More case studies and testing are needed before these ecological principles can be used to make predictions more generally about different climate and toxicant combinations or effects in different ecosystems. The levels of complexity associated with community ecotoxicology, combined with the multitude of potential climate change impacts, means that a list of knowledge gaps for different species and ecosystems would be endless. Here, we instead focus on research approaches that, in our view, are particularly promising for investigating ecological impacts of contaminants under climate change and on further developments that are needed.

Shifting baselines or reference conditions due to climate change are recognized as a great challenge in ERA and restoration ecology 17. A potential method for separating the ecological effects of local anthropogenic stressors from the effects of a regional climatic trend is analysis of long-term data series from contaminated and reference sites in the same region. However, climatic trends are generally slow compared to the high temporal variation; therefore, such analysis would require longer time series than are generally available in ecotoxicology (see *Case study 2*). Prediction of the most likely climate and toxicant interactions will therefore require integration of modeling, experiments, and field investigations. Studies of regular large-scale climatic forcing, such as the North Atlantic Oscillation and the El Niño–Southern Oscillation, have given much insight into climatic influences on ecological processes (reviewed in Stenseth et al. 89). Such short-term for long-term approaches would also bring more realism into field studies of toxicant effects under climate change 38. Nevertheless, the time frame of ecological responses to these climatic phenomena would still be short compared with the long-term climatic trends. Space-for-time approaches (see *Case study 3*) can serve as a replacement for long-term investigations, letting a warmer climate zone represent future climatic conditions, for example. However, even if a spatial environmental trend can be a good proxy of future environmental conditions, the populations along this spatial gradient may not be representative of populations experiencing such conditions in the future. The populations along the spatial gradient may be well adapted to the current conditions, whereas a future environmental change is more likely to cause suboptimal conditions.

Micro- and mesocosm experiments are clearly important tools for studying combined impacts of climate and toxicant stressors, although there is disagreement and ongoing debate concerning the modeling methods for analysis and interpretation of such data 90–92. Long-term ecotoxicological experiments that incorporate a realistic downscaling of future climate projections in combination with high environmental variation would enable more reliable predictions of toxicant impacts under climate change. Within such systems, long-term stabilizing and escalating processes could be assessed and used as a basis for ecological models. Research on microevolutionary responses to GCC and toxicants requires careful study designs, and to this end, the following three approaches are promising (see also De Schamphelaere et al. 61): (1) studies of adaptive potential and trade-offs, using genetic variability and correlation analyses; (2) microevolution experiments; and (3) comparing the climatic tolerance of reference populations and populations resistant to toxicants.

Ecological models are being used increasingly in population and community ecotoxicology 93; but in general, more development and validation are needed for practical applications in risk assessment. Models are also being used extensively in predicting climate change impacts on population dynamics, species distributions, and biodiversity (e.g., 94). So far, however, there has been little integration of climate change and ecology in ecotoxicological modeling. We recommend that future development in population- and community-level modeling focus on one or more of the following aspects: nonadditive interactions between climatic stressors and toxicants, capacity for compensation or feedback mechanisms at the population and community levels, potential for recovery, potential for adaptation to stressors, and spatial dynamics. We expect that more descriptions of relationships between species traits and climate change vulnerability will be reported in the near future (e.g., http://www.climate-and-freshwater.info/). We therefore envision that the trait-based framework developed for assessing population vulnerability to toxicants 74 can be expanded to assess population vulnerability to combined toxicant and climate stress. Using a combination of these trait-based approaches, one could identify which species will be particularly vulnerable to this combination of stressors under different climate scenarios. More generally, Akçakaya et al. 94 suggested that predictions of climate change impacts on biodiversity can be improved through consideration of interactions among habitat shifts, landscape structure, and demography for a number of species, using a combination of models. They stated that this approach might allow the development of guidelines for assigning species to threat categories, based on a combination of life-history parameters, characteristics of the landscapes in which they live, and projected range changes. In our view, incorporating assessment of species' vulnerability due to current and future contaminant exposure would further improve these predictions.
